# 

**Published:** 1881-11

**Authors:** 


VOL. II. NOVEMBER, 1881. NO. II.

—: THE :—

### The Independent Practitioner:

INDEXED

A MONTHLY JOURNAL,

DEVOTED TO

### Medical, Surgical, Obstetrical, Dental, Hygienical and Popular Sciences.

EDITED BY

BASIL M. WILKERSON, D. D. S., M. D.

**"Altera poscit opem res, et conjurat amice."**

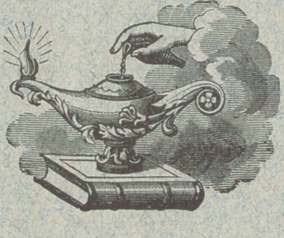

**NEW YORK:**

*Published by B. M. WILKERSON, Proprietor.*

Office, 20 Conrtlandt Street.

**$3.00 Per Annum in Advance, - - Single Copies 30 Cents.**

ENTERED AT THE POST OFFICE, NEW YORK CITY, AS SECOND-CLASS MATTER.

